# Quality properties of puffed corn snacks incorporated with sesame seed powder

**DOI:** 10.1002/fsn3.532

**Published:** 2017-11-20

**Authors:** Fataneh Hashempour‐Baltork, Mohammadali Torbati, Sodeif Azadmard‐Damirchi, Geoffrey P. Savage

**Affiliations:** ^1^ Department of Food Science and Technology Faculty of Nutrition Tabriz University of Medical Sciences Tabriz Iran; ^2^ Department of Food Science and Technology Faculty of Agriculture University of Tabriz Tabriz Iran; ^3^ Food Group Department of Wine, Food and Molecular Biosciences Lincoln University Canterbury New Zealand

**Keywords:** Antioxidant, essential fatty acid, extrusion, oxidation stability, puffed corn snack, sesame powder

## Abstract

Puffed corn snacks are tasty and affordable cereal‐based food products which have a relatively poor nutritional quality. Sesame seed is a rich source of essential amino and fatty acids, phenolic compounds, tocopherols, and antioxidants. In this study, puffed corn snacks were produced by incorporating sesame powder at 0% (control sample), 5%, 10%, and 15% levels in its formulation and stored at room temperature (24°C) for 60 days. Fatty acid composition, tocopherol, sesamin and sesamolin, phenolic compounds, peroxide value (PV), acidity (AV), and sensory evaluation of the samples were determined. The results indicate that oleic acid content increased and palmitic acid decreased significantly (*p* < .05) in all the samples at 10% and 15% inclusion levels. The content of phenolic compounds, γ‐tocopherols, sesamin, and sesamolin had significant increases in all the formulated samples. PV results indicated that the formulated samples had a higher stability when the ratio of sesame powder was increased, while the AV values showed a significant increase during storage. Incorporation of 10% sesame powder in the snack formulation had a positive effect on the stability, sensory, and nutritional quality of the product. This approach can be used to modify nutritional quality of this food product and introduce to food market as a relatively healthy snack.

## 1 INTRODUCTION

Extrusion is one of the most important new processes used in modern food technology; its application has increased in the past two decades (Li, Zhang, Jin, & Hsieh, 2005). Extrusion is an advantageous, cleaner, and more effective technology which can yield a product with the same or even better quality than traditional technology products (Brncic et al., 2009). This process has been used in the production of many varieties of foods including snack foods, breakfast cereals, and other ready‐to‐eat textured foods (Brncic et al., 2009).

Snack foods are tasty and affordable products which are available in various types and are appreciated by consumers in every age group. Extruded puffed corn snacks exist in the snack group of products and are made using corn grits, rice, wheat, or other cereals, and are often flavored with cheese, oil, chili, onion or garlic powder, and many other spices. However, health concerns cause many consumers to reduce the consumption of extruded snacks in their diets because many of these products contain 25% oil which contains saturated fatty acids and also a high content of *trans* fatty acids. On the other hand, their major ingredients are cereals which contain low protein and essential amino acid contents, particularly lysine (Majumdar & Singh, 2014).

Various efforts have been made to produce snacks with higher nutritional and lower adverse effects by incorporating different additional sources such as soy protein isolate (Veronica, Olusola, & Adebowale, 2006), soy flour (Nwabueze, 2007), shrimp powder (Shaviklo, Azaribeh, Moradi, & Zangeneh, 2015), tomato powder (Huang, Peng, Lu, Lui, & Lin, 2006), soy flour and oat bran (Lobato, Anibal, Lazaretti, & Grossmann, 2011), different flour and powder (Alam, Pathania, & Sharma, 2016; Pęksa et al., 2016; Rodríguez‐Miranda et al., 2011; Wang, Zhang, & Mujumdar, 2012), yam flour (Alves & Grossmann, 2002), and vitamins (Athar et al., 2006).

Sesame seed (*Sesamum indicum* L.) has gained considerable attention in human nutrition in the production of functional foods (Abou‐Gharbia, Shehata, & Shahidi, 2000). Sesame seed is a rich source of essential amino acids, essential fatty acids, tocopherols, and phenolic compounds including sesamol, sesamolin, phenylethanethiol, vinylguaiacol, furyl‐methanthiol, and furaneol (Alobo, 2001; Moazzami & Kamal‐Eldin, 2006). These compounds lead to high oxidation stability of sesame seed oil (Hashempour‐Baltork, Torbati, Azadmard‐Damirchi, & Savage, 2016). This seed contains a high amount of minerals and B‐complex vitamins such as thiamin, niacin, folic acid, riboflavin, and pyridoxine (Garavand & Madadlou, 2014). Furthermore, the consumption of sesame seed can decrease the risk of cardiovascular diseases and certain cancers (Fremont, Belguendouz, & Delpal, 1999).

Effects of temperature and screw speed of extruder has been investigated on the physical properties of corn extrudates containing sesame seed, but there is no information about its effect on oxidation stability of the final product (Nikmaram, Garavand, Elhamirad, Beiraghi‐toosi, & Goli‐movahhed, 2015). Therefore, the present study covers the evaluation of the physical, chemical, and sensory properties of puffed corn snacks containing sesame seeds and defines the optimum level of its addition to introduce a new nutritious and tasty products. This research is a preliminary study on the shelf life and quality assessment of puffed corn snack stored for up to 60 days. However, it should be mentioned that further researches are required for long‐term assessment.

## 2 MATERIALS AND METHODS

### 2.1 Materials

Corn grit and white sesame seed were purchased from the central Pirnia grocery market (Tehran, Iran) and were cleaned by handpicking and winnowing to remove foreign matter before they were milled to a fine powder using a Moulinex mill grinder (Type 320, made in EC). In this study, all chemicals were analytical grade and purchased from Sigma Chemical Co. (St. Louis, MO).

### 2.2 Formulation

Sesame powder was added to the ingredients of the puffed corn snack (corn flour [63.5%], vegetable oil [26%], sunset yellow colored cheese powder [6.85%], milk powder [2.15%], and salt [1.5%]) at levels of 0% (control sample), 5%, 10%, and 15% of total content of ingredient and were produced using an extrusion process.

### 2.3 Extrusion cooking

A twin screw extruder (DS56‐III; Jinan Saixin, China) was used in this study. The extruder properties included die diameter 3.6 mm, barrel diameter 640 mm, and ratio of length to diameter (L:D) 12:1. The temperature of the feeding section, mixing part, and exit section were adjusted to 50, 100, and 140°C, respectively. The puffed corn snacks after production were cooled to room temperature (24°C), and were then packed in 100 mm × 100 mm × 40 μm polyethylene bags (with two layers). The samples were then kept at (24°C) for up to 60 days.

### 2.4 Experiments

Fatty acid composition, tocopherol, sesamin, and sesamolin contents, and also sensory analysis of samples were analyzed on the 1st and on the 60th days. Phenolic compounds, peroxide value (PV), and acidity (AV) were evaluated on the production day and every 15 days.

### 2.5 Oil extraction

The fatty acid composition, tocopherol, sesamin, sesamolin, peroxide, and acid values were determined on the oil extracted at room temperature from the processed samples using the method described by Conkerton, Wan, and Richard (1995).

### 2.6 Fatty acid composition

Fatty acids methyl ester were prepared using the ISO 1995 method and analyzed by gas chromatograph (GC‐1000, DANI, Italy), and flame ionization detector was slightly modified according to Azadmard‐Damirchi and Dutta (2006). The identification of fatty acids was carried out by obtaining chromatograms and comparing their retention times with standard fatty acid methyl esters (Sigma Aldrich, St. Louis, MO).

### 2.7 Phenolic compounds

Phenolic compounds were determined using the method described by Capannesi, Palchetti, Mascini, and Parenti (2000) which uses the Folin–Ciocalteu reagent, and gallic acid was used for the calibration curve and results were reported as mg gallic acid/kg of oil.

### 2.8 Sesamin and sesamolin

Sesamin and sesamolin were determined by HPLC according to the method described by Moazzami, Andersson, and Kamal‐Eldin (2006).

### 2.9 Tocopherols

Tocopherol determination was performed according to the method described by Fathi‐Achachlouei and Azadmard‐Damirchi (2009) using HPLC (KNAUER, German) equipped with a column packed with 5 μm LiChrosorb SI (250 mm × 4.6 mm) and florescence detector (RFKNAUER‐10XL). α‐ and γ‐Tocopherols of puffed corn snack samples were determined with reference to standard retention times.

### 2.10 Peroxide and acid values

Peroxide and acid values of the puffed corn snack oils were measured using the AOAC (2000, 2005) methods, respectively.

### 2.11 Expansion ratio

Expansion of the snack samples was measured using the AACC, 2000 (10–10B) method.

### 2.12 Sensory evaluation

Sensory evaluation was performed by a panel of 30 male and female semitrained assessors. A 5‐point hedonic scale (1 = *extremely dislike* and 5 = *extremely like*) was used to determine the appearance attributes including color, aroma, taste, crispiness, and overall acceptability.

### 2.13 Statistical analysis

Analysis of variances (ANOVA) in factorial experiments in completely randomized design by 16.0 SPSS was used as the statistical software (Chicago, IL) for analyzing the obtained data. Duncan's multiple range post hoc test was used to analyze the significant differences at 0.05 level.

## 3 RESULT AND DISCUSSION

### 3.1 Fatty acid composition

In addition to other ingredients, vegetable oils are also used in puffed corn snack formulations. It was possible to use sesame seed oil instead of its powder in the formulation as well. However, there are some bioactive and nutritional compounds such as amino acids, vitamins, phenolic compounds, fibers, and other hydrophilic components in sesame seeds (Alobo, 2001; Garavand & Madadlou, 2014; Hashempour‐Baltork et al., 2016) that remains mostly in cake during oil extraction. Therefore, in this study, sesame seed powder has been used instead of its oil to make puffed corn snack more nutritious.

Sesame seed oil has high content of linoleic acid and oleic acid followed by palmitic acid and stearic acid (Gharby et al., 2015; Yoshida & Takagi 1997). Addition of sesame powder to puffed corn snack materials caused significant changes (*p* < .05) in the fatty acid profile (Table 1). Palmitic and linolenic acids were decreased by increasing sesame powder content, because these fatty acids are low in sesame powder (Codex, 2003). Because of the high level of oleic acid in sesame seed, increasing the percentage of sesame powder in formulation leads to a significant increase (*p* < .05) in oleic acid in the processed samples. On the other hand, in the formulated product, stearic acid and linoleic acid did not show a significant difference (*p* < .05) from the control sample which is related to the amount of these fatty acids in sesame seed and oil used in the puffed corn snack formulation (Codex Alimentarius, 2003).

**Table 1:** Fatty acid composition (%) of puffed corn snacks incorporated with sesame seed powder on the first day and after 60 days of storage

Sample^a^	16:0		18:0		18:1		18:2		18:3	
	Day 1	Day 60	Day 1	Day 60	Day 1	Day 60	Day 1	Day 60	Day 1	Day 60
Control	16.01 ± 0.07	17.04 ± 0.08	4.05 ± 0.06	5.03 ± 0.19	24.7366 ± 0.97	23.336 ± 0.25	48.0166 ± 0.11	44.92 ± 1.25	2.02 ± 0.078	1.05 ± 0.11
5%	14.94 ± 0.1	14.946 ± 0.04	4.01 ± 0.12	5.08 ± 0.13	27.723 ± 0.16	26.526 ± 0.65	46.81 ± 0.26	42.03 ± 1.5	1.006 ± 0.066	1.053 ± 0.21
10%	12.12 ± 0.21	13.016 ± 0.11	4.04 ± 0.05	4.02 ± 0.25	32.52 ± 0.07	30.04 ± 0.08	46.723 ± 0.31	42.0433 ± 2.1	1.01 ± 0.1	1.08 ± 0.13
15%	11.12 ± 0.08	12.103 ± 0.15	4.053 ± 0.08	4.059 ± 0.07	34.941 ± 0.22	34.03 ± 0.52	46.043 ± 0.23	40.03 ± 1.30	1.0133 ± 0.10	1.053 ± 0.08

Analysis of variance	*df*	Fatty acid composition (%)
Sample	3	**
Fatty acid	4	**
Storage	1	**
Sample × Fatty acid	12	**
Sample × Storage	3	*
Fatty acid × Storage	4	**
Sample × Fatty acid × Storage	12	**
LSD sample		0.24
LSD fatty acid		0.26
LSD storage		0.17
LSD sample × Fatty acid		0.53
LSD sample × Storage		0.33
LSD fatty acid × Storage		0.37
LSD sample × Fatty acid × Storage		0.75

a Treatments were control, 5%, 10%, and 15% samples with 0%, 5%, 10%, and 15% of sesame seed powder, respectively.

All values are the mean of three replicates ± *SD* of the mean.

LSD, least significant difference.

**p < .01, *p < .05.

Fatty acid composition of the oils can be reduced by oxidation during storage. Due to the high stability of saturated fatty acids, these fatty acids did not show a significant change (*p* < .05) during 2 months of storage. However, generally there was a significant decrease (*p* < .05) in the level of linolenic acid during storage. These results are in agreement with previous reports which showed that fatty acid composition changes during storage (Table 1) (Gomez‐Alonso, Mancebo‐Campos, Salvador, & Fregapane, 2007).

### 3.2 Total phenolic compounds

**Figure 1:**
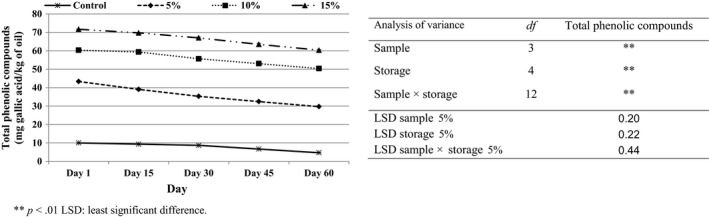
Total phenolic compounds of puffed corn snacks incorporated with sesame seed powder during storage

Phenolic compounds have important roles in nutritional, oxidation stability, and organoleptic qualities of food products (Nergiz & Unal, 1991). Increasing the contents of sesame powder, which contains a high level of phenolic compounds showed a significant increase (*p* < .05) in the total phenolic compounds (TPC) of the formulated samples (Figure 1).

A significant (*p* < .05) decrease in the TPC content occurred after 2 months of storage. This decrease can be related to oxidation and hydrolytic activities which occur because of the effects of temperature, oxygen, and enzymes during storage (Cinquanta, Esti, & La Notte, 1997). Because powdered sesame seeds were used during the manufacture of these products, the surface to volume ratio was increased, exposing more of the product to air, and therefore the oxidation rate and phenolic compound loss were increased.

### 3.3 Sesamin and sesamolin

Sesamin and sesamolin are the potent phenolic antioxidants which are abundant in sesame seed (Hashempour‐Baltork et al., 2016). As expected, the control sample did not contain any sesamin and sesamolin, but the formulated puffed corn snacks contained increased levels of these compounds. As expected, the 5%, 10%, and 15% samples had a significant increase (*p* < .05) in the amount of sesame powder in the final product (Table 2).

**Table 2:** Sesamin, sesamolin, and α‐ and γ‐tocopherol content (mg/kg) of puffed corn snacks incorporated with sesame seed powder on 1st and 60th days of storage

Sample	Sesamin		Sesamolin		α‐Tocopherol		γ‐Tocopherol	
	Day 1	Day 60	Day 1	Day 60	Day 1	Day 60	Day 1	Day 60
Control	ND	ND	ND	ND	350.35 ± 4.5	309.70 ± 1.4	230.36 ± 2.4	230.03 ± 0.9
5%	15.30 ± 0.21	14.46 ± 0.17	10.25 ± 0.25	10.043 ± 0.25	329.36 ± 4.06	305.02 ± 4.9	280.67 ± 1.15	275.02 ± 0.9
10%	38.25 ± 0.25	35.93 ± 0.12	19.716 ± 0.21	8.36 ± 0.15	319.36 ± 4.03	305.36 ± 0.56	310.02 ± 0.99	310.02 ± 1.98
15%	61.51 ± 0.5	58.31 ± 0.12	27.2 ± 0.18	24.84 ± 0.26	314.37 ± 2.06	300.05 ± 5.9	320.02 ± 1.9	310.02 ± 2.01

Analysis of variance	*df*	Sesamin and sesamolin contents
Sample	2	**
Sesamin and sesamolin	1	**
Storage	1	**
Sample × Sesamin and sesamolin	2	**
Sample × Storage	2	*
Sesamin and sesamolin × Storage	1	**
Sample × Sesamin and sesamolin × Storage	2	**
LSD sample		0.21
LSD sesamin and sesamolin		0.17
LSD storage		0.17
LSD sample × Sesamin and sesamolin		0.30
LSD sample × Storage		0.30
LSD sesamin and sesamolin × Storage		0.24
LSD sample × Sesamin and sesamolin × Storage		0.42

Analysis of variance	*df*	α‐ and γ‐Tocopherol contents
Sample	3	**
Tocopherol	1	**
Storage	1	**
Sample × Tocopherol	3	**
Sample × Storage	3	**
Tocopherol × Storage	1	**
Sample × Tocopherol × Storage	3	**
LSD sample		2.56
LSD tocopherol		2.09
LSD storage		1.81
LSD sample × Tocopherol		3.62
LSD sample × Storage		3.62
LSD tocopherol × Storage		2.56
LSD sample × Tocopherol × Storage		5.12

ND, not detected; LSD, least significant difference.

For treatments, see Table 1. All values are the mean of three replicates ± *SD* of the mean.

**p < .01, *p < .05.

The levels of sesamin and sesamolin were significantly reduced (*p* < .05) following storage for 60 days at room temperature (Table 2). The levels of sesamolin in the product were more reduced than sesamin, which is reported previously as well (Fukuda, Nagata, Osawa, & Namiki, 1986).

Earlier researches showed that sesamols were more effective at increasing the stability of oils than tocopherol and they can also have a positive synergetic action with γ‐tocopherol (Yoshida & Takagi, 1997, 1999).

### 3.4 Tocopherols

α‐ and γ‐Tocopherols are important vitamins in foods and they have significant antioxidant properties in many food products. Many studies have reported that temperature, oxygen, light, and storage period are the main factors that lead to the reduction of vitamins during storage (Hidalgo, Brandolini, & Pompei, 2009). Fortification of puffed corn snack with sesame powder caused a significant increase in γ‐tocopherol content, but with a relatively low reduction in α‐tocopherol content of produced product. Results from this study indicate that a significant decrease (*p* < .05) in α‐tocopherol occurred during storage, but γ‐tocopherol was much more stable during storage (Table 2).

A significant decrease (*p* < .05) in the level of α‐tocopherol occurred during storage even when the sesame powder was increased in the puffed corn snack (Table 2); similar reductions in the γ‐tocopherol content also occurred during storage. These results are in agreement with sesame seed component because total tocopherol in virgin sesame seed oil is reported to be 500 mg/kg oil with the γ‐tocopherol and δ‐tocopherol consisting of 90% and 7% of the total amounts, respectively. Furthermore, sesame seed contains a very high level of α‐tocopherol (Gharby et al., 2015).

### 3.5 Peroxide value

Oxidation of fatty acids leads to the generation of peroxides which can have an adverse effect on health and the quality of oils and also foods containing these oils. The processing and storage temperatures and porosity of puffed corn snacks are the main factors which can lead to increasing rates of oil oxidation.

**Figure 2:**
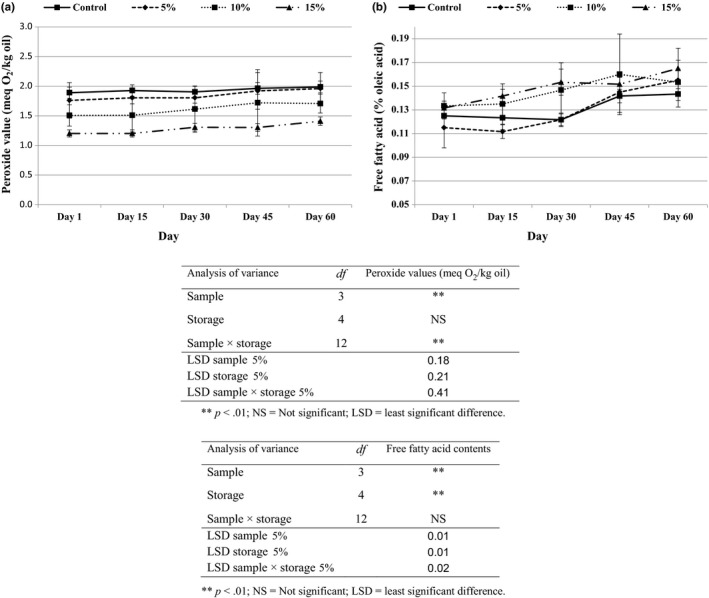
(a) Peroxide values (meq O_2_/kg oil) and (b) free fatty acid contents (% oleic acid) of puffed corn snacks incorporated with sesame seed powder during storage. For treatments see Table 1

In this research, all samples showed a slight increase in PV during storage (Figure 2a). Snack samples containing sesame powder had lower PVs, and increasing the content of sesame in the product improved its stability. The PV of the sample containing 5% sesame powder was not significantly different (*p* < .05) from the control sample, but samples with 10% and 15% showed a significant decrease (*p* < .05) in PV (Figure 2a). The content of lignan and natural antioxidants in sesame seed powder is known to improve the oxidative stability of puffed corn snack.

### 3.6 Acidity value

Acidity in processed food is known to be an important parameter which is related to hydrolysis of triacylglycerols and generation of free fatty acids in oils (Pereira, Casal, Bento, & Oliveira, 2002). The results indicate that all samples had a significant increase (*p* < .05) in acid value during storage. Samples containing higher levels of sesame powder had higher acidity (Figure 2b); however, these amounts were too low to make them unfavorable. The acidity values should not be higher than 4.0 mg KOH/g oil to be unacceptable in foods (Codex Alimentarius, 2003). It have been previously reported that hydrolysis by lipase enzymes, increased temperature, light, and moisture are the main factors in forming free fatty acids (Pereira et al., 2002). Milling oil seeds leads to an increase in the exposure of oil and lipase in foods and can increase the AV in samples.

### 3.7 Expansion ratio

**Figure 3:**
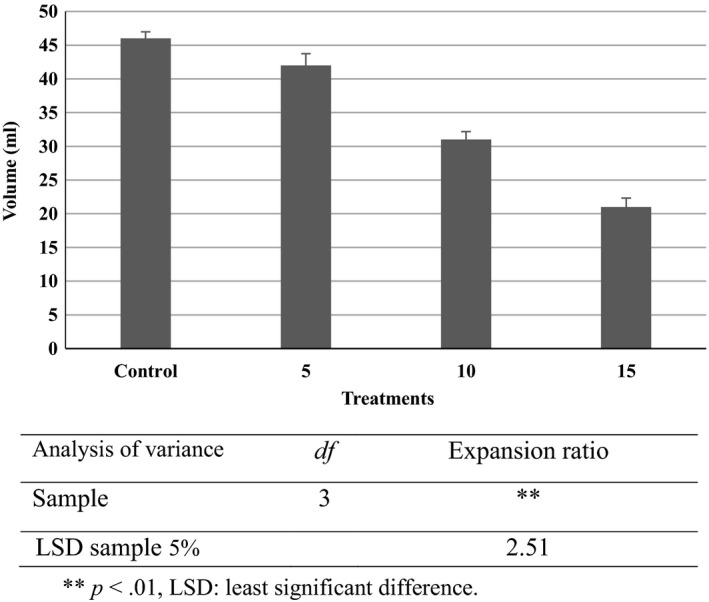
Expansion ratio of puffed corn snacks incorporated with sesame seed powder. For treatments see Table 1

Expansion of puffed corn snacks is a desired attribute for consumers and industry. Expansion ratio of extrudates with 0%–15% sesame powder varied from 21 to 45 mL in a 5‐g sample (Figure 3). Addition of sesame powder significantly reduced (*p* < .05) the expansion ratio of the snacks. High melting viscosity of ingredients leads to the retention of air bubbles in the starch matrix (Nascimento, Carvalho, Takeiti, Freitas, & Ascheri, 2012). Fat, protein, and dietary fiber which are found in sesame seed can decrease the ingredient viscosity and in turn decrease the product expansion. On the other hand, the existing protein can surround the starch and reduce the expansion ratio (Onwulata, Smith, Konstance, & Holsinger, 2001). The results from this study confirm the earlier reports on the effects of the sesame oil cake on extrudate expansion (Nascimento et al., 2012). Also, it has been observed that foxtail millet flour which contains higher amounts of dietary fiber content leads to a reduction in the expansion ratio of extrudates (Deshpande & Poshadri, 2011). Protein enrichment materials lead to an increase in the density of snacks which can cause the reduction in its expansion on processing (Veronica et al., 2006).

### 3.8 Sensory analysis

Organoleptic properties of food products are one of the most important factors in accepting and choosing them for consumption. Sensory evaluation of these products indicate that the samples with 5% and 10% sesame powder had the highest score from flavor and aroma point of views. The panelists gave the highest crispness score to control sample of the products and when the content of sesame powder was increased, the acceptability of the product was decreased. The effect of sesame powder level on the overall acceptability of puffed corn snack indicated that samples with 5% and 10% had the good acceptance, but higher amounts of sesame powder in the formulation led to reduction in product acceptability (Table 3).

**Table 3:** Sensory evaluation of puffed corn snacks incorporated with sesame seed powder

	Control^a^		5%		10%		15%	
	Day 1	Day 60	Day 1	Day 60	Day 1	Day 60	Day 1	Day 60
Flavor	3.36 ± 0.03	3.27 ± 0.02	4.62 ± 0.01	4.53 ± 0.01	4.14 ± 0.02	4.06 ± 0.03	3.42 ± 0.01	3.45 ± 0.01
Smell	4.06 ± 0.03	3.98 ± 0.03	4.74 ± 0.02	4.73 ± 0.03	4.68 ± 0.04	4.68 ± 0.06	4.34 ± 0.02	4.3 ± 0.01
Crispness	4.72 ± 0.01	4.69 ± 0.02	4.52 ± 0.01	4.57 ± 0.03	4.02 ± 0.01	3.98 ± 0.03	2.32 ± 0.01	2.37 ± 0.04
Volume	5.08 ± 0.04	5.04 ± 0.05	4.74 ± 0.02	4.68 ± 0.01	4.64 ± 0.02	4.69 ± 0.06	3.86 ± 0.03	3.86 ± 0.04
Appearance	4.72 ± 0.01	4.84 ± 0.08	4.74 ± 0.07	4.67 ± 0.05	4.60 ± 0.05	4.5 ± 0.02	2.52 ± 0.01	2.58 ± 0.03
Color	3.14 ± 0.02	3.11 ± 0.01	4.06 ± 0.03	4.13 ± 0.04	3.8 ± 0.05	3.81 ± 0.04	3.72 ± 0.01	3.70 ± 0.02

Analysis of variance	*df*	Sensory evaluation
Attribute	5	**
Sample	3	**
Storage	1	**
Attribute × Sample	15	**
Attribute × Storage	5	**
Sample × Storage	3	**
Attribute × Sample × Storage	15	**
LSD attribute		0.01
LSD sample		0.01
LSD storage		0.00
LSD attribute × Sample		0.02
LSD attribute × Storage		0.01
LSD sample × Storage		0.01
LSD attribute × Sample × Storage		0.02

a For treatments, see Table 1.

All values are the mean of three replicates ± *SD* of the mean.

LSD, least significant difference.

**p < .01.

## 4 CONCLUSION

The obtained results indicate that enrichment of puffed corn snacks with sesame seed powder is an alternative for improving its nutritional effects and converting the puffed corn snack to a potential functional food. Addition of sesame powder leads to a significant increase (*p* < .05) in the level of phenolic compounds, tocopherols, sesamin, and sesamolin in the formulated products. Physicochemical evaluation of samples showed that using sesame powder in the formulation of puffed corn snack leads to an increase in the oxidation stability of the samples during storage. Also, sensory evaluation indicated that samples containing 5%–10% of sesame powder were more acceptable in taste tests. In conclusion, results obtained show that it is possible to incorporate sesame seed powder in the formulation of puffed corn snack and introduce a new food product to the market.
